# The demographic and socioeconomic correlates of behavior and HIV infection status across sub-Saharan Africa

**DOI:** 10.1038/s43856-022-00170-z

**Published:** 2022-08-18

**Authors:** Chirag J. Patel, Kajal T. Claypool, Eric Chow, Ming-Kei Chung, Don Mai, Jessie Chen, Eran Bendavid

**Affiliations:** 1 Department of Biomedical Informatics, Harvard Medical School, Boston, MA USA; 2 Lincoln Laboratory, MIT, Lexington, MA USA; 3 Rosalind Franklin University School of Medicine, Chicago, IL USA; 4 Alphabet Inc., Mountain View, CA USA; 5 Loyola University, Chicago, IL USA; 6 Center for Population Health Sciences, Stanford University, Stanford, CA USA; 7 Department of Health Policy, Stanford University, Stanford, CA USA

**Keywords:** HIV infections, Epidemiology

## Abstract

**Background:**

Predisposition to become HIV positive (HIV + ) is influenced by a wide range of correlated economic, environmental, demographic, social, and behavioral factors. While evidence among a candidate handful have strong evidence, there is lack of a consensus among the vast array of variables measured in large surveys.

**Methods:**

We performed a comprehensive data-driven search for correlates of HIV positivity in >600,000 participants of the Demographic and Health Survey across 29 sub-Saharan African countries from 2003 to 2017. We associated a total of 7251 and of 6,288 unique variables with HIV positivity in females and males respectively in each of the 50 surveys. We performed a meta-analysis within countries to attain 29 country-specific associations.

**Results:**

Here we identify 344 (5.4% out possible) and 373 (5.1%) associations with HIV + in males and females, respectively, with robust statistical support. The associations are consistent in directionality across countries and sexes. The association sizes among individual correlates and their predictive capability were low to modest, but comparable to established factors. Among the identified associations, variables identifying being head of household among females was identified in 17 countries with a mean odds ratio (OR) of 2.5 (OR range: 1.1–3.5, R^2^ = 0.01). Other common associations were identified, including marital status, education, age, and ownership of land or livestock.

**Conclusions:**

Our continent-wide search for variables has identified under-recognized variables associated with being HIV + that are consistent across the continent and sex. Many of the association sizes are as high as established risk factors for HIV positivity, including male circumcision.

## Plain language summary

Sub-Saharan Africa has a high prevalence of HIV. It is important to know what individuals are at risk of becoming HIV-positive (HIV + ), so that testing resources and therapy, if HIV + , can be prioritised to control HIV transmission. The risk of becoming HIV + may be driven by many socioeconomic and behavioral factors. Here, we analyzed a large dataset with information about these factors in over 300,000 HIV + or HIV-negative people from 29 Sub-Saharan African countries. We identify over 300 factors that are associated with HIV positivity, such as wealth, capital ownership, and number of unions. Some of these factors have not yet been well studied, such as marital status, while others are well known preventative measures, such as male circumcision. Any one factor plays but a small role in HIV positivity, and this study highlights many possible potential routes for prevention.

## Abstract (web summary)

Patel et al. perform a comprehensive analysis of factors associated with HIV positivity across 29 sub-Saharan African countries from 2003 to 2017. They identify over 300 factors associated with HIV positivity in each sex, some of them under-recognized but consistent across countries and sex.

## Introduction

The number of new HIV infections in Sub-Saharan Africa has been declining for over a decade, but the prevalence of HIV has stagnated, around 1 million people are newly infected every year, and over 5 million are unaware of being HIV-positive^1,2^. The global strategy for improving the population burden of HIV calls for intensive identification of HIV-infected individuals in order to pursue high treatment coverage, which, in turn, is anticipated to reduce HIV transmission and incidence^1^. Sub-Saharan Africa–where the burden of HIV is highest – remains short of these major targets, including identifying at least 90% of those with HIV^1^. One challenge hindering progress at identifying those with HIV infection is the heterogeneity of HIV risk in terms of modes of transmission and prevalence. In other words, it is hard to know where and whom to target for testing and public health interventions when aiming for very high (>90%) identification rates. This heterogeneity is manifest in the proliferation of inquiries into HIV risk factors, without unifying approaches or systematic studies into HIV risk^3–7^. At the time of this writing, no systematic reviews of HIV risk factors in Africa have been published. In this paper we describe a systematic approach to the identification of a broad array of behavior, economic, and social factors in HIV risk and across multiple samples and geographic regions of Africa. The goal of this analysis is to help with identifying groups that may benefit from HIV-specific public health efforts.

The epidemiology of risk factor identification commonly proceeds by identifying candidate risk factors. These approaches are grounded in real-world observations but suffer from several limitations: generalizing to populations other than those directly examined is often tenuous; testing of one or several candidate risk factors may spuriously identify candidate risk factors;^8,9^ and limited number of candidate risk factors may leave important relationships unobserved^10^.

We address these shortcomings using a large-scale risk factor testing approach across the entire sample of individuals that had an HIV test as part of the Demographic and Health Surveys (DHS) between 2003 and 2017 in Sub-Saharan Africa, a sample of over 700,000 individuals extending our previous work to identify factors associated with HIV in Zambian females^11^. We test every variable or candidate risk factor available in DHS against individual-level HIV outcomes, a total of 32,353 variables (27,506 in females and 24,713 in males). This approach allows us to systematically identify variables that include behavioral, social, and economic factors and have a high degree of generalizability as risk factors for HIV, that play an important role in explaining who does and does not have HIV, over multiple survey waves across multiple countries in Sub-Saharan Africa, in men and women separately.

## Methods

### Data sources

The primary data for this work comes from the Demographic and Health Surveys (DHS). The DHS are nationally representative surveys conducted approximately every 5 years in many low- and middle-income countries. We used data from every individual who had consented and tested positive or negative for HIV-1^12^. There were a total of 50 surveys available for analysis for females and males each across 29 countries. We followed the STROBE statement and checklist in preparation and analysis of the data and approval for the use of the data was approved by the DHS program.

According to the DHS Program, procedures and questionnaires for standard DHS surveys have been reviewed and approved by ICF (the administrator of the DHS Survey) Institutional Review Board (IRB). Second, country-specific DHS survey protocols are reviewed by the ICF IRB as well as country-specific IRB. ICF IRB ensures that the survey complies with the U.S. Department of Health and Human Services regulations for the protection of human subjects (45 CFR 46). Further, before each interview, an informed consent statement is read to the participant, who can then decline or accept the interview. The informed consent statement emphasizes that participation is voluntary; that the respondent may refuse to answer any question, and information will be kept confidential. More information can be found on the DHS Program website: https://dhsprogram.com/Methodology/Protecting-the-Privacy-of-DHS-Survey-Respondents.cfm.

### Appending the women and men’s recode

While appending the individual and men’s recode datasets (IR and MR), we also systematically identified common variables to map between MR and IR. The following prefix changes were made to variables in MR in this sequence: removing “_”, “mv” to “v”, removing “s”, “mcase” to “case”, “m” to “v”, “sm” to “mm” or “s” if no “mm” candidates exist, “dm” to “d”, and “sm6” to “s8” and “mv304a” to “v304a_”. These changes allowed us to harmonize variables with similar content across men's and women's surveys.

Additionally, IR variables that had at least a 70% syntactic similarity to MR variables (computed using Levenshtein distance) were mapped using Amazon Mechanical Turk (mTurk) Master Workers. These workers were trained briefly and presented with an MR variable and a set of potential IR candidates. They selected the IR candidate that they thought was the best match, or indicated that the MR variable was an individual category of the question header label, or that none of the IR candidates were appropriate. We validated the mTurk responses by excluding any workers who only selected the same positional answer (or semantically similar answers), or whose responses were not submitted through the mTurk interface. A majority vote rule (70%) was used to select the MR-IR variable mapping, and any candidate mappings that were indeterminate remained unmapped.

### Harmonizing the household, male, and female surveys

Each DHS contains several parts: a census of all household members, in-depth interviews with reproductive-age (15–49 years old) women, men (15–59 years old), a ledger of household information, and HIV test results for each tested individual. We harmonized the surveys to match individuals in the household with available in-depth data, household data, and HIV test results. Variables that represented the same concept were identified by similarities in their variable name or the variable description, and merged together. Amazon Mechanical Turk (mTurk) was used to match men’s and women’s variables when the relationship between the variable names was intermediate^13^. A harmonized dataset was created for each country-wave survey, and each of these merged surveys was then analyzed.

We analyzed the harmonized surveys from all sub-Saharan African countries and survey years that performed the DHS HIV testing protocol. All surveys that reported an HIV prevalence ≥ 0.01 were included in our analysis (see Table 1).

**Table 1:** Sample sizes across sub-Saharan Africa.

Country	Sample Size	# Cases	Prevalence (SE)	Prevalence Female (SE)	Prevalence Male (SE)	Mean Age (SE)	% Female (SE)	% Urban (SE)
Angola	12260	271	1.95 (0.2)	2.57 (0.28)	1.21 (0.22)	27.71 (0.16)	54.21 (0.57)	70.77 (2.41)
Burkina_Faso	23434	295	1.17 (0.09)	1.29 (0.11)	1.04 (0.13)	29.1 (0.1)	53.93 (0.33)	26.43 (1.85)
Burundi	8790	167	1.43 (0.15)	1.72 (0.19)	1.11 (0.17)	27.75 (0.15)	52.78 (0.54)	12.55 (1.62)
Cameroon	10348	565	5.35 (0.3)	6.75 (0.44)	3.91 (0.31)	28.63 (0.12)	50.5 (0.49)	56.23 (2.68)
Chad	11034	172	1.56 (0.17)	1.8 (0.23)	1.29 (0.21)	28.11 (0.16)	52.73 (0.53)	25.74 (2.36)
Congo_Democratic_Republic	27530	313	1.17 (0.13)	1.62 (0.2)	0.7 (0.11)	28.96 (0.13)	51.76 (0.39)	39.74 (2.36)
Congo	12382	379	3.16 (0.3)	4.12 (0.46)	2.06 (0.28)	28.95 (0.18)	53.16 (0.42)	63.07 (3.91)
Cote_d'Ivoire	18070	721	4.33 (0.24)	5.49 (0.34)	3.11 (0.28)	27.82 (0.16)	51.15 (0.52)	49.44 (3.19)
Ethiopia	41165	787	1.43 (0.12)	1.86 (0.17)	0.98 (0.1)	28.31 (0.09)	51.48 (0.28)	20.79 (1.97)
Gabon	11264	502	4.24 (0.38)	5.81 (0.6)	2.69 (0.36)	28.65 (0.18)	49.67 (0.64)	87.65 (1.74)
Ghana	18795	351	1.96 (0.13)	2.56 (0.2)	1.31 (0.15)	29.89 (0.11)	51.79 (0.38)	49.62 (2.04)
Guinea	15407	272	1.69 (0.14)	2.03 (0.2)	1.27 (0.14)	29.71 (0.13)	55.12 (0.39)	36.11 (2.42)
Kenya	13359	902	6.54 (0.39)	8.32 (0.48)	4.63 (0.4)	28.8 (0.12)	51.78 (0.5)	24.71 (2.15)
Lesotho	18730	4308	23.67 (0.47)	27.57 (0.59)	18.99 (0.57)	28.66 (0.1)	54.52 (0.45)	29.57 (1.76)
Liberia	20362	320	1.65 (0.15)	1.88 (0.18)	1.39 (0.2)	29.37 (0.15)	54.04 (0.42)	48.69 (2.74)
Malawi	34409	3514	10.25 (0.31)	12.13 (0.39)	8.24 (0.33)	28.37 (0.08)	51.61 (0.28)	18.92 (1.18)
Mali	17978	199	1.16 (0.12)	1.36 (0.15)	0.93 (0.14)	29.58 (0.12)	53.25 (0.38)	30.21 (2.33)
Mozambique	16976	1466	7.48 (0.47)	8.75 (0.56)	5.97 (0.47)	32.49 (0.22)	54.43 (0.4)	31.03 (3.53)
Namibia	9309	1297	14.33 (0.6)	16.84 (0.75)	11.45 (0.81)	32.69 (0.23)	53.51 (0.72)	54.62 (2.64)
Niger	7897	70	0.63 (0.1)	0.58 (0.12)	0.7 (0.15)	30.31 (0.16)	57.32 (0.6)	22.55 (2.38)
Rwanda	41044	1206	2.8 (0.11)	3.38 (0.15)	2.17 (0.11)	28.8 (0.07)	52.28 (0.21)	17.15 (1.24)
Sao_Tome_and_Principe	4860	78	1.54 (0.25)	1.29 (0.29)	1.79 (0.35)	29.77 (0.21)	50.49 (0.92)	52.15 (5.62)
Senegal	18122	123	0.52 (0.07)	0.57 (0.11)	0.43 (0.12)	28.37 (0.11)	53.66 (0.73)	52.62 (2.53)
Sierra_Leone	21619	328	1.46 (0.12)	1.68 (0.15)	1.22 (0.16)	29.69 (0.13)	52.87 (0.29)	36.29 (2.36)
Swaziland	13008	2481	18.89 (0.58)	22.25 (0.69)	15 (0.67)	26.89 (0.12)	53.69 (0.53)	23.02 (2.47)
Tanzania	44896	2180	5.8 (0.19)	6.69 (0.24)	4.75 (0.22)	28.45 (0.06)	54.18 (0.26)	26.84 (1.54)
Togo	9321	200	2.5 (0.22)	3.11 (0.3)	1.85 (0.24)	29.34 (0.14)	51.65 (0.52)	44.81 (3.05)
Uganda	31644	1592	5.2 (0.21)	6.02 (0.27)	4.26 (0.22)	30.65 (0.11)	53.4 (0.31)	18.05 (2.08)
Zambia	40821	5634	13.68 (0.37)	15.34 (0.45)	11.97 (0.39)	28.68 (0.07)	50.7 (0.26)	45.19 (2.04)
Zimbabwe	43835	7158	15.71 (0.3)	18.35 (0.37)	12.76 (0.33)	28.01 (0.06)	52.8 (0.31)	35.71 (1.61)

### Resolving value-label conflicts

When mapping categorical variables together in IR and MR, we resolved conflicting value labels by: 1) obtaining a complete list of all possible values of categorical variables, 2) merging all labels to the values if value-labels are available in the native Stata data dictionary, 3) appending both complete lists of value-labels from IR and MR, 4) removing duplicates from this new value-label dictionary, 5) finding conflicting value-labels (where the same value in a variable has two different labels, e.g. 1 = “Yes” in IR but 1 = “Definitely” in MR) and re-levelling one of the conflicting value-labels. If a conflict is found, then the value without a label receives priority (and the labelled conflict is relevelled); otherwise the IR value keeps its level while the MR value gets relevelled to the next larger level number in the set of levels in that variable. All value-labels are prefixed with their original value in square brackets in the label (e.g. the relevelled value of 2 might have the label “[1] Yes” because the value of 1 had two conflicting labels).

### Merging recode datasets

The household member recode (PR) is then left-joined with the appended IR/MR file (with variables mapped and levels relevelled) in a 1:1 merge using (1) the cluster ID (v001), (2) household ID (v002), (3) respondent line number (v003), and occasionally, (4) structure ID (sstruct, sconces, svivi, sqnumber, or snumber) if it is needed to uniquely identify a respondent. Not all PR records are merged to a record in the appended IR/MR file, but are retained in the dataset with empty cells for the IR/MR variables. The HR dataset is left-joined to the PR/IR/MR dataset in a 1:m merge using (1) the cluster ID, and (2) household ID. The AR dataset (which contains the HIV test result of a subset of respondents) is left-joined to the PR/IR/MR/HR dataset in a 1:1 merge and left as missing if not available. In the AR file, v001 = hivclust, v002 = hivnumb and v003 = hivline. The GE dataset is left-joined in a 1:m merge using cluster ID only.

### Handling missing recode datasets

The IR dataset is the minimum file needed for merging. If the IR dataset is not present, the datasets are not merged. Alerts are given if <75% of the IR/MR records are merged to PR, or if >25% of the IR/MR records are not mergeable to PR. Similarly, alerts are given if <75% of HR records are merged to PR, or if >25% of HR records are not mergeable to PR. Alerts are given if <50% of HIV test result data are merged to PR. The final merged dataset of PR, IR/MR, HR, and AR was saved as a Stata 15 file with the embedded data dictionary and named after the country-survey code and given the “flattened” suffix.

### Processing of duplicate and categorical variables

We removed variables that exhibited no variation or were “duplicates”. We then computed pairwise Pearson correlations for all variables in a given survey across all 50 surveys. We identified groups of variables whose pairwise Pearson correlation was 0.9 or greater. For each of these groups of variables that were highly correlated with one another, we selected one to represent the group that had the largest average sample size for the variable across all surveys. These decision rules eliminated redundant variables, preserved most meaningfully continuous variables, and discretized most non-ordinal variables. All continuous variables were scaled and centered.

### Selection and preparation of variables per survey

We retained all variables that had at least 90% complete data, a total of 29,092 and 25,980 unique variables in female surveys and male surveys, respectively, and 33,729 unique variables overall in both males and females. All variables with 30 or fewer levels were treated as binary variables. Variables with 30 or more levels were treated as continuous. We retained 7251 and 6288 unique variables in females and males, respectively and 8980 unique variables overall. The number of unique variables per country ranged from 337 (Congo) to 1659 (Malawi) for females and from 348 (Congo) to 1219 (Malawi) for males.

### Number of surveys available per country

Five countries had 3 surveys available for analysis (Lesotho, Malawi, Rwanda, Tanzania, Zimbabwe), 11 had 2 surveys (Burkina Faso, Democratic Republic of Congo, Cote d'Ivoire, Ethiopia, Ghana, Guinea, Kenya, Liberia, Mali, Sierra Leone, Zambia). The 13 remaining countries had 1 survey (Angola, Burundi, Cameroon, Chad, Congo, Gabon, Mozambique, Namibia, Sao Tome and Principe, Senegal, eSwatini, Togo, and Uganda).

### Pan Sub-Saharan sex-specific systematic meta-analyses across all years and countries

For each variable, we combined associations across all of the surveys (year and country combination) for males and females with a random effects meta-analysis procedure. Specifically, given an association between a variable (e.g., marital status, call it *X*1) and HIV + in a survey for a sex (e.g., Zambia, 2013-2014, females denoted by ${\beta }_{{Zambia},2013\mbox{--}2014,{females}}^{1}$; Zimbabwe, 2005, females, denoted by ${\beta }_{{Zambia},2005,{females}}^{1}$), we estimated the overall association for each of the variables and measures of their heterogeneity, including the I^2^ for males and females using a DerSimonian-Laird random effects meta-analysis model, arriving at an overall estimate of association (e.g., for our example,${\beta }_{{females}}^{1}$)^14^. Out of the 7,251 and 6,288 variables unique available for females and males, 2,830 and 2,307 variables for females and males were measured in greater than one survey and thus available for a meta-analysis (Supplementary Fig. 1A, B). As examples, 4421 and 3921 variables were only available in 1 survey for males and females respectively. 79 and 57 variables were available across all 50 surveys for females and males respectively. For the 4421 and 3921 variables that appeared only in one survey (for females and males), we retained the survey-specific estimate. We used the *metafor* package in R to compute the meta-analytic association estimates^15^.

### Survey-specific association models and meta-analysis across sub-Saharan Africa

We associated each of the variables with HIV status using a weighted logistic regression model:

$${logit}({{HIV}}_{{sj}})=\alpha +{\beta }_{s}^{i}{X}_{{sj}}^{i}.$$

Where *s* indexes the survey (including country, year, and female or male survey; e.g., Zimbabwe, 2005, female survey), *j* indicates the individual observation (all analyses are person-level analyses), and *i* indexes the variables for that survey. We estimated the Nagelkerke pseudo-R^2^ to assess the improved goodness of fit from a logistic model with zero variables (equivalent to the prevalence of HIV) to a model with *X*^16^. We used survey-weighted logistic regression model to account for the probability-based sampling of DHS, implemented in the *survey* package in R^17^. In a senstivity analysis, we tested the associations in a multivariate model with adjustments for age, place of residence (urban or rural), and the DHS wealth index (5 point scale of relative wealth). Last, for each variable, we combined associations across all of the surveys (year and country combination) for males and females with a random effects meta-analysis procedure, estimating the average association and heterogeneity of the associations across the surveys and countries.

We prioritized variables that were the most explanatory and whose associations were statistically non-zero across the surveys after correction for multiple hypotheses. Specifically, we report variables across pan-Saharan-Africa meta-analytic p-value lower than a conservative DHS-wide Bonferroni threshold of 1 × 10^−6^ (for 7,251 plus 6,288 variables, a Bonferroni threshold would be 0.05/13,539, or *p* < 3.7 × 10^−6^) and whose average R2 across the surveys were the top 25% of all R2 for males and females, which was equivalent to a R2 of 0.001.

### Sex-specific meta-analyses within countries

For each variable, we combined associations of all of the surveys within a country (e.g., Zambia, 2007 and Zambia 2013-2014) to estimate a country-specific association for each sex. Given an association between a variable (i.e, marital status, call it *X*1) and HIV+ in a survey for a sex (i.e, Zambia, 2013–2014, females denoted by ${\beta }_{{Zambia},2013\mbox{-}2014,{females}}^{1}$ in the equation; Zambia, 2007, females, denoted by ${\beta }_{{Zambia},2007,{females}}^{1}$), we estimated the overall association for each of the variables for males and females using a DerSimonian-Laird random effects meta-analysis model, arriving at an overall estimate of association (${\beta }_{{Zambia},{females}}^{1}$).

### Comparison of associations assayed across multiple countries

**Fig. 1:**
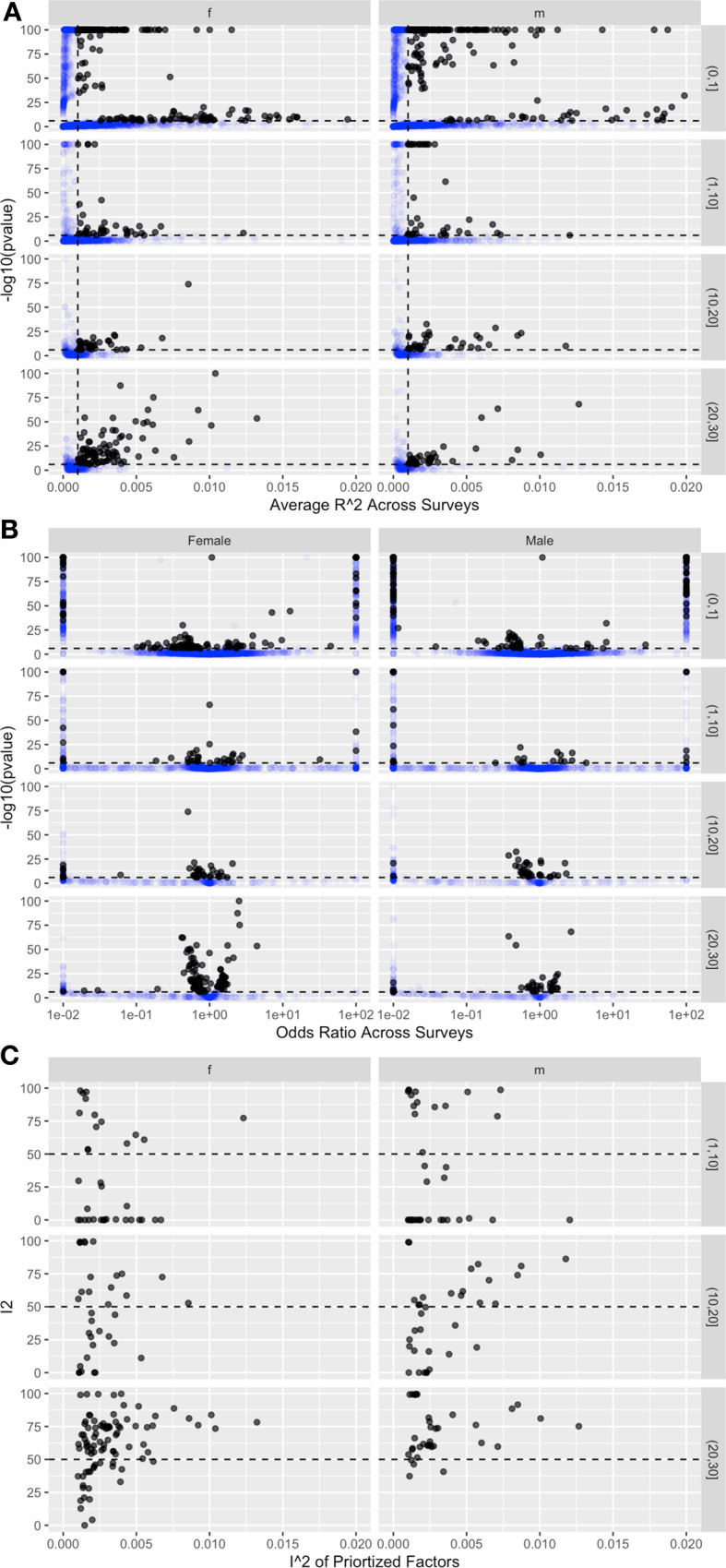
Volcano plots of -log10(*p* value) versus R^2^ and Odds Ratios. **A** Nagelkerke R^2^ (**B**) Odds Ratios (**C**) Heterogeneity I^2^ of identified variables across all surveys. OR capped at 0.01 and 100 for visualization purposes. In **A** and **B**, Dotted line denotes Bonferroni-level of significance (1 x 10^−6^). In **C**, dotted line corresponds to I^2^ of 50%. f females. m males.

To facilitate comparison of associations that are measured in multiple countries, we binned by the number of countries in which the variables appear, including 1 country, 2–10 countries, 11–19 countries, and 20–29 countries (Fig. 1, Fig. S2A–C, Table 2). Variables that were identified and assessed across a larger number of countries exhibited similar Nagelkerke R^2^ distributions (Fig. 1A, Fig. S2A); however, their odds ratios were attenuated (Fig. 1B, Fig. S2B).

**Table 2:** Distributions of odds ratios, Nagelkerke R^2^, and I^2^ (heterogeneity) estimates across countries.

gender/number	Num Assoc.	25th OR	Median OR	75th OR	25th R2	Median R2	75th R2	25th I2	Median I2	75th I2
**1 Country**										
Female	4543	1.2	1.62	3.69	8.56 × 10^−5^	3.58 × 10^−4^	1.17 × 10^−3^	.	.	.
Male	4089	1.24	1.78	12.67	8.39 × 10^−5^	3.29 × 10^−4^	1.12 × 10^−3^	.	.	.
**2–10 Countries**										
Female	1702	1.19	1.67	136.4	2.56 × 10^−4^	6.30 × 10^−4^	1.39 × 10^−3^	0	62.1	99.01
Male	1488	1.24	2.3	526.3	2.42 × 10^−4^	5.91 × 10^−4^	1.26 × 10^−3^	0	64.85	99.14
**11–20 Countries**										
Female	467	1.15	3.93	110.95	4.10 × 10^−4^	7.67 × 10^−4^	1.22 × 10^−3^	49.8	98.59	99.39
Male	368	1.17	3.44	189.43	4.09 × 10^−4^	7.70 × 10^−4^	1.57 × 10^−3^	47.49	98.38	99.41
**20–29 Countries**										
Female	539	1.17	1.65	9.88	4.77 × 10^−4^	8.42 × 10^−4^	1.42 × 10^−3^	56.04	97.57	99.35
Male	343	1.26	3.68	56.47	4.71 × 10^−4^	7.10 × 10^−4^	1.22 × 10^−3^	67.7	99.14	99.48

Num Assoc. denotes the number of associations for that category.

### Multivariate association study

The primary analyses in this project are univariate. This was done to maintain the tractability of the analysis, as multivariate analyses can increase the dimensionality and reduce sample sizes of the analysis substantially. However, we recognize that univariate associations, even those with very tight associations and meaningful effect sizes, can be confounded. We thus present the findings of a multivariate analysis with a fixed choice of covariates, replicating only the basic association study where all variables are examined and assessed as in the primary survey-specific analysis.

We run the following model on all candidate variables:

$${logit}({{HIV}}_{{sj}})=\alpha +{\beta }_{s}^{i}{X}_{{sj}}^{i}+{\delta }_{s}^{k}{{WEALTH}}_{{sj}}^{k}+{\varsigma }_{s}{{AGE}}_{{sj}}+{\lambda }_{s}{{RURAL}}_{{sj}}$$

The indexing is identical to the model described, and we additionally include 3 variables: wealth (a 5-point relative wealth based on household assets, indexed by *k*), age in years, and place of residence (using an indicator for living in a rural area). As with the univariate analyses, the ${\beta }_{s}^{i}$ are combined across surveys and countries using a random-effects meta-analysis.

The results of the meta-analyzed multivariate analysis are presented in Supplementary Data 5-6, and a histogram comparing the OR values of the univariate and multivariate models are in Figure S3.

### Prediction of HIV+ across aub-Saharan Africa

We calculated the predicted probability of HIV as a function of the variables that were identified in common in 29 African countries. First, we identified the latest survey with HIV status for each country and then built a logistic regression model for the top 10 identified factors (had *p* value less than 1 × 10^−6^ and R2 greater than 0.001) present in all 29 countries. We removed DHS participants who did not have all 10 variables available. Using this model, we estimated the area under the curve and the predicted probability for each individual.

The Area Under the Curve (AUC) estimates were calculated by varying the threshold at which a person was considered “HIV+” based on their predicted probability, estimating the sensitivity and specificity based on these thresholds, and calculating the area under the receiver operator curve (AUC). Precision-recall curves are preferred when the proportion of cases are much lower than non-cases by focusing on positive cases and are estimated by computing the area under the precision (positive-predictive value) versus recall (sensitivity) curve (PRAUC). For random predictors, the AUC will be equal to approximately 0.5 and the PRAUC will be equal to the prevalence of HIV+. Therefore, a predictor that achieves an AUC greater than 0.5 or a PRAUC greater than the prevalence is considered to be better than random. A perfect AUC or PRAUC is 1.

Finally, we assess the concentration of HIV risk. We do this by calculating the proportion of the population that carries the predicted probability of HIV from our model. This allows us to calculate a “gini” coefficient of HIV risk by estimating the cumulative HIV risk distribution that is accounted for by the portion of the sample population using the *ineq* package in R^18^. For example, the gini coefficient allows us to see if 20% of HIV risk is accounted for by 80% of the population as ranked by predicted risk^19^.

### Statistics and reproducibility

Data preparation was performed using Stata 15 MP on a MacBook Pro with a 2.7 GHz Intel Core i7 processor and 16GB of RAM. In Stata 15, setting the maxvar to 18000 (or higher) is necessary given the high number of variables. The codebase for data preparation is available on Zenodo (10.5281/zenodo.6819777)^20^.

The source code for meta-analysis across surveys is available here: https://github.com/chiragjp/dhs_hiv_meta. A website of the findings is here: https://www.chiragjpgroup.org/dhs_hiv_meta/. We have placed all of our summary statistics, including the overall and country-specific sample sizes and meta-analytic odds ratios, R^2^, I^2^, standard errors, and p-values in Supplementary Data 1–4. All summary statistics for all surveys can be found on Zenodo (see reference^20^).

### Reporting summary

Further information on research design is available in the Nature Research Reporting Summary linked to this article.

## Results

### Cohort characteristics

We harmonized 50 DHS surveys from 29 African countries conducted between 2003 and 2017, in which 619,468 participants were tested for HIV (47% male, 6.1% positive) (Table 1). While we only analyzed surveys with HIV prevalence at or above 1% of the adult population, the variability in the prevalence levels ranged from 1% to 25% (Table 1). As described in the methods, there were 7,251 and 6,288 unique variables in females and males respectively (Fig. S1).

### Distribution of associations across sub-Saharan Africa points to consistency in direction but variability in size of associations

Overall, we found robust but heterogenous associations between behavioral, social, environmental, behavioral variables with HIV+ across the 29 countries of sub-Saharan Africa (Fig. 1A–C, Fig. S2 [for single country], Table 2). In the following, we call “identified variables” as those that had a Bonferroni-level p-value less than 1 × 10^−6^ and a Nagelkerke R^2^ of greater than 0.001. Each of the estimated overall and country-specific odds ratios, standard errors, and pvalues are documented in extensive online content (Supplementary Data 1–4).

Many of the identified variables spanned behavioral, social, biological, economic, and environmental domains and exhibited consistent direction of association across Africa, the heterogeneity in risk magnitude and I^2^ was large (Fig. 1, Table S1, Table 2). For identified variables with associations in at least 2 surveys, the median odds ratio (in absolute value) was 1.73 (IQR: 1.18 to 73.83) and 2.6 (IQR: 1.24 to 263.09) for females and males respectively. Associations across at least 2 surveys were highly heterogeneous and had a median I^2^ of 75% (IQR: 17.6 to 99.3) and 81.9 (IQR: 17.8 to 99.2) for females and males (Table S1). The median Nagelkerke R^2^ among females was 3.6x10-4 (IQR: 8.6x10^−5^ to 1.17x10^−3^), and among males the median R^2^ was 3.29x10^−4^ (IQR: 8.39x10x^−5^ to 3.29x10^−5^) (Table S1).

### Variables identified across sub-Saharan Africa

We identified 344 (5.4% out of 6,288 possible) and 373 (5.1% out of 7251 possible) variables in their association with HIV+ (R^2^ greater than 0.001 and p-value less than 1 × 10^−6^) in males and females respectively (Table 2). For variables that were surveyed among female participants in 11–19 countries (a total of 432 variables), we identified 35 variables (8.1%). Second, for variables that were surveyed among female participants in 20–29 countries (a total of 449 variables), we identified 90 variables (20%). Third, in the male sample, we identified 38 (2.6% out of 1450), 34 (10% out of 334), 36 (11.7% out of 307) variables. A total of 168 and 154 variables were assessed in all 29 countries for females and males. Of these, we identified 31 (18%) and 16 (10%) in females and males respectively. Among the 31 identified variables assessed in all 29 countries in females, the median R^2^ was 2.9 × 10^−3^, median odds ratio was 1.57, and the I^2^ was 69.43%. For the 16 identified variables assessed in all 29 countries in males, the median R^2^ was 2.5 × 10^−3^, median odds ratio was 1.39, and median I^2^ was 60.6%. The distribution of the R2 were comparable for variables assayed in different number of countries (Fig. 1A), while variables with extreme odds ratios diminished when appearing in larger number of countries (Fig. 1B). Variables broadly exhibited high heterogeneity in their odds ratios across countries (Fig. 1C), with a majority having I^2^ greater than 50%.

**Fig. 2:**
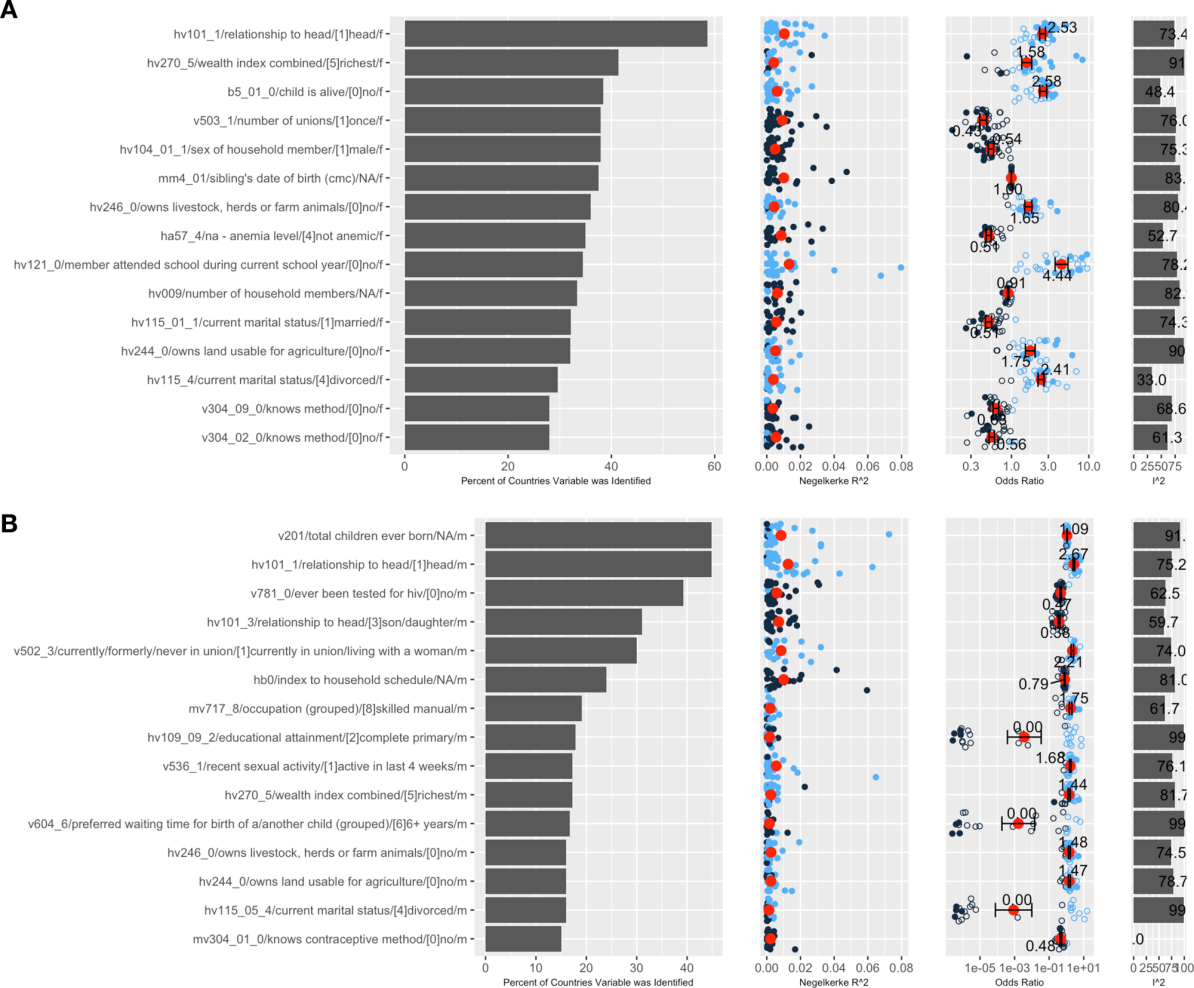
Top 15 variables identified across 21–29 countries. **A** Top 15 variables in females and (**B**) Top 15 variables in males. Left most panel is the variable name and code name, 2nd panel from left is the percent of countries variable was identified, 3rd panel from left is the Nagelkerke R2 per country (red dot is the average; blue points are countries with OR > 1, dark blue are countries with OR < 1), 4th panel shows the odds ratios (overall meta-analytic estimate in the red dot), and 5th panel shows the I2 (heterogeneity). Error bars denote 2 times the standard error of the coefficient, which was estimated using a random-effects model. All data to reproduce figures, including the standard errors, can be found in Supplementary Data 1. The sample sizes (number of surveys) for the overall odds ratio (red) are in Supplementary Data 1.

**Fig. 3:**
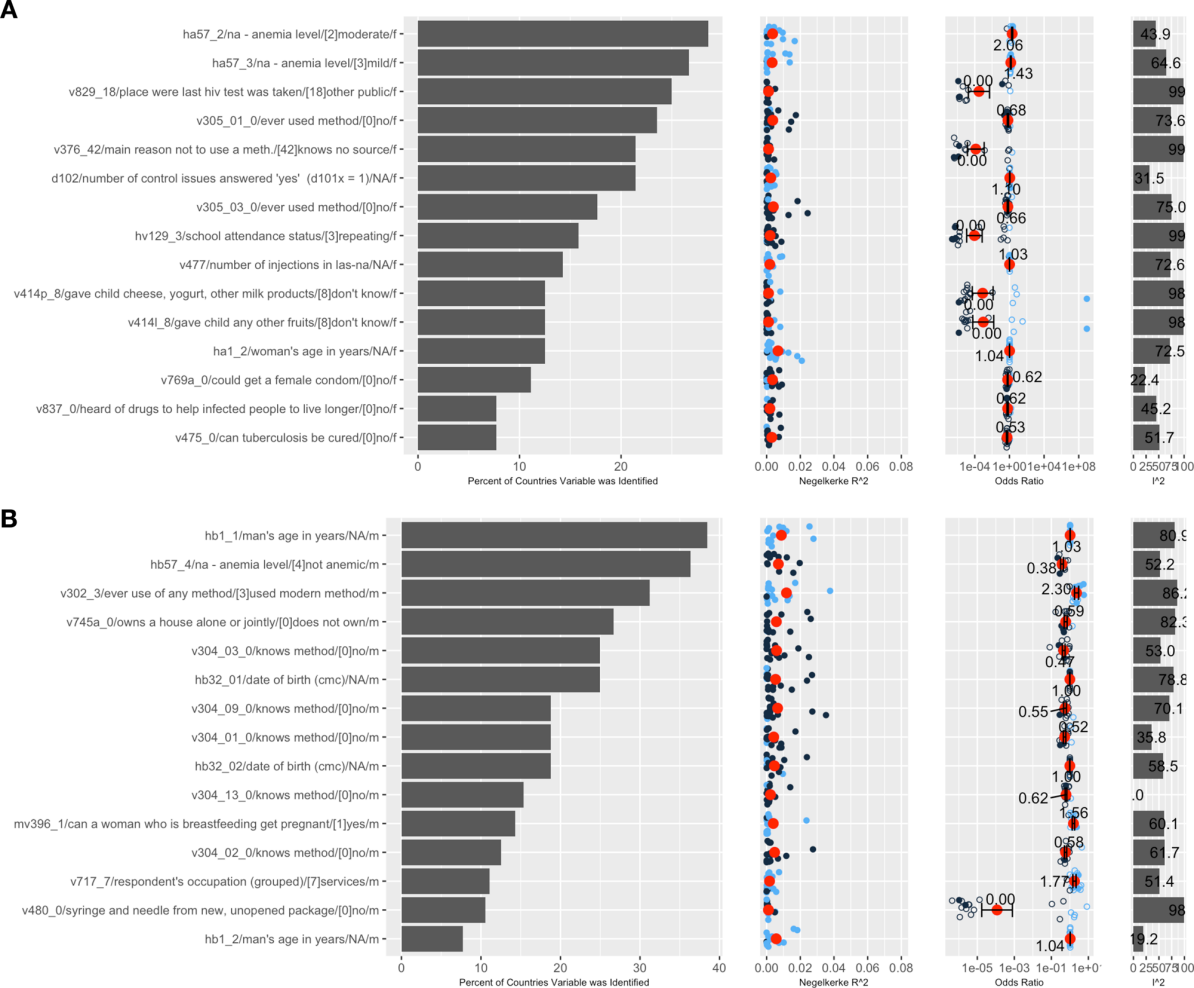
Top 15 variables identified across 11–20 countries. **A** Top 15 variables in females and **B** Top 15 variables in males. Left most panel is the variable name and code name, 2nd panel from left is the percent of countries variable was identified, 3rd panel from left is the Nagelkerke R2 per country (red dot is the average; blue points are countries with OR > 1, dark blue are countries with OR < 1), 4th panel shows the odds ratios (overall meta-analytic estimate in the red dot), and 5th panel shows the I2 (heterogeneity)). Error bars denote 2 times the standard error of the coefficient, which was estimated using a random-effects model. All data to reproduce figures, including the standard errors, can be found in Supplementary Data 2. The sample sizes (number of surveys) for the overall odds ratio (red) are in Supplementary Data 2.

We highlight several variables that show a striking relationship with HIV+ (Figs. 2 and 3). The variable indicating women who are the head of the household was significantly associated with HIV in nearly 60% of study countries, explained 1% of the variation in HIV status on average (and nearly 4% in two countries), and was uniformly associated positively with HIV, with a meta-analytic odds ratio of 2.5 (2.5 increased odds for HIV+ relative to females who were not the head of the household) (min-max 1.1–3.5) for all countries (Fig. 2A, first row). The analogous indicator, head of household among men, was identified in 40% of countries, explaining as much as 6% of the variability in HIV status, and was also positively associated with HIV+ status (Fig. 2B, 2nd row). As above, the heterogeneity in the odds ratios for females and males was 75%. In other words, there was substantial variability in the odds ratios.

Other indicators of marital status were associated with HIV+ across the subcontinent, including the number of unions, if the participant had been married, and if they were divorced among females. First, females who had been in one union had 60% decreased odds across all countries in the sub-continent to be HIV+ and were identified in slightly less than 40% of the countries (range of the Nagelkerke R^2^ was .1% to 4%, Fig. 2A, 4th row). Second, if a woman was divorced, they had a consistently increased odds (median OR 2.4) in HIV+ relative to those not divorced. This was consistent throughout the study countries (I^2^ estimate of 33%).

Males who were divorced had an inconsistent association with HIV+ (I^2^ 99%) across countries. Specifically, in 13 out of 28 countries divorced males had a near zero chance of HIV+ (Fig. 2B, 14th line from top, see ORs <1 × 10^−5^). These countries included Sao Tome and Principe, Kenya, Tanzania, Democratic Republic of Congo, Sierra Leone, Togo, Gabon, Chad, Senegal, Ghana, Guinea, Burkina Faso, and Rwanda. For the other countries, their associations had lower and insignificant association sizes (filled point in figure). Third, males who were in a union (or living with) a woman at the time of the survey had an average odds ratio of 2.2, or greater than 2-fold increase in odds versus relative to those who were not for HIV+, and this was consistent across the study countries, with Nagelkerke R^2^ reaching up to 4% (Fig. 2B, 5th row). Of note, marital status also stood out in our previous analysis of HIV+ in Zambia^11^.

Complex indicators of education status emerged as a key correlate in numerous countries. For example, we report a meta-analytic OR of 4.4 across 29 countries for the female participant not attending school (Fig. 2A). While this indicator may overlap with young age, low educational attainment was also associated with higher risk of HIV among men (Fig. 2B). Similarly, the meta-analytic OR across 28 countries for males who completed primary school versus who did not was extremely small, 0.004 (Fig. 2B, 8^th^ row from the top) in 12 out of 28 countries, indicating near zero chance of HIV+. Countries with near-zero number of males with HIV+ that have completed primary school included Namibia, Mozambique, Kenya, Gabon, Uganda, Ghana, Angola, Rwanda, Ethiopia, Guinea, Mali, and Congo.

We found several variables that reflected potential biological and co-morbid conditions with HIV+ (Fig. 3A, B). For example, we identified men who were not anemic had an overall 60% decreased odds of HIV+ (with Nagelkerke R^2^ up to 4%). This variable was found in 25% of the countries it was measured (Fig. 3B).

The possibility of confounding with some of the identified variables was observed in the adjusted analyses (Supplementary Data 5 and 6). We note two patterns from the adjusted analyses. First, the large majority of the variables identified in the univariate analyses remained significantly associated with HIV status in the adjusted analyses. Second, the effect size of those variables was, on the whole, smaller in the adjusted analyses. We elaborate on the implications of these findings in the Discussion.

### Prediction of HIV status

**Fig. 4:**
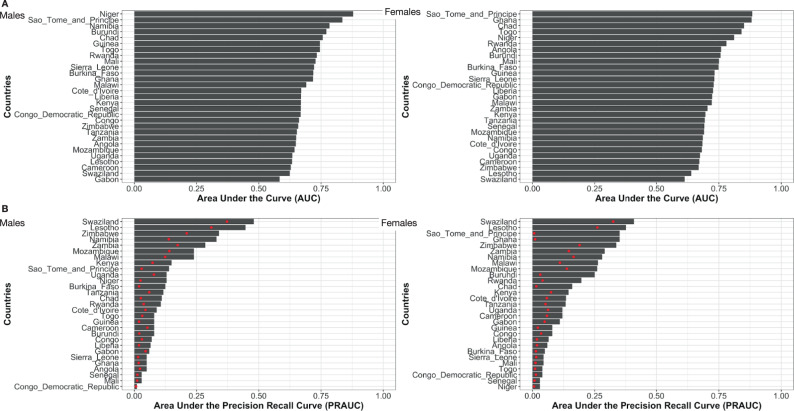
Predictive capability of top 10 variables. **A** Area under the receiver-operator-curve (ROC) and **B** Are under the precision-recall curve. Left panels are prediction for males and right panel for females. Red points in **B** denote the prevalence of HIV+. Sample sizes for cases and controls for each AUC and precision-recall curve calculation are in Table S2.

**Fig. 5:**
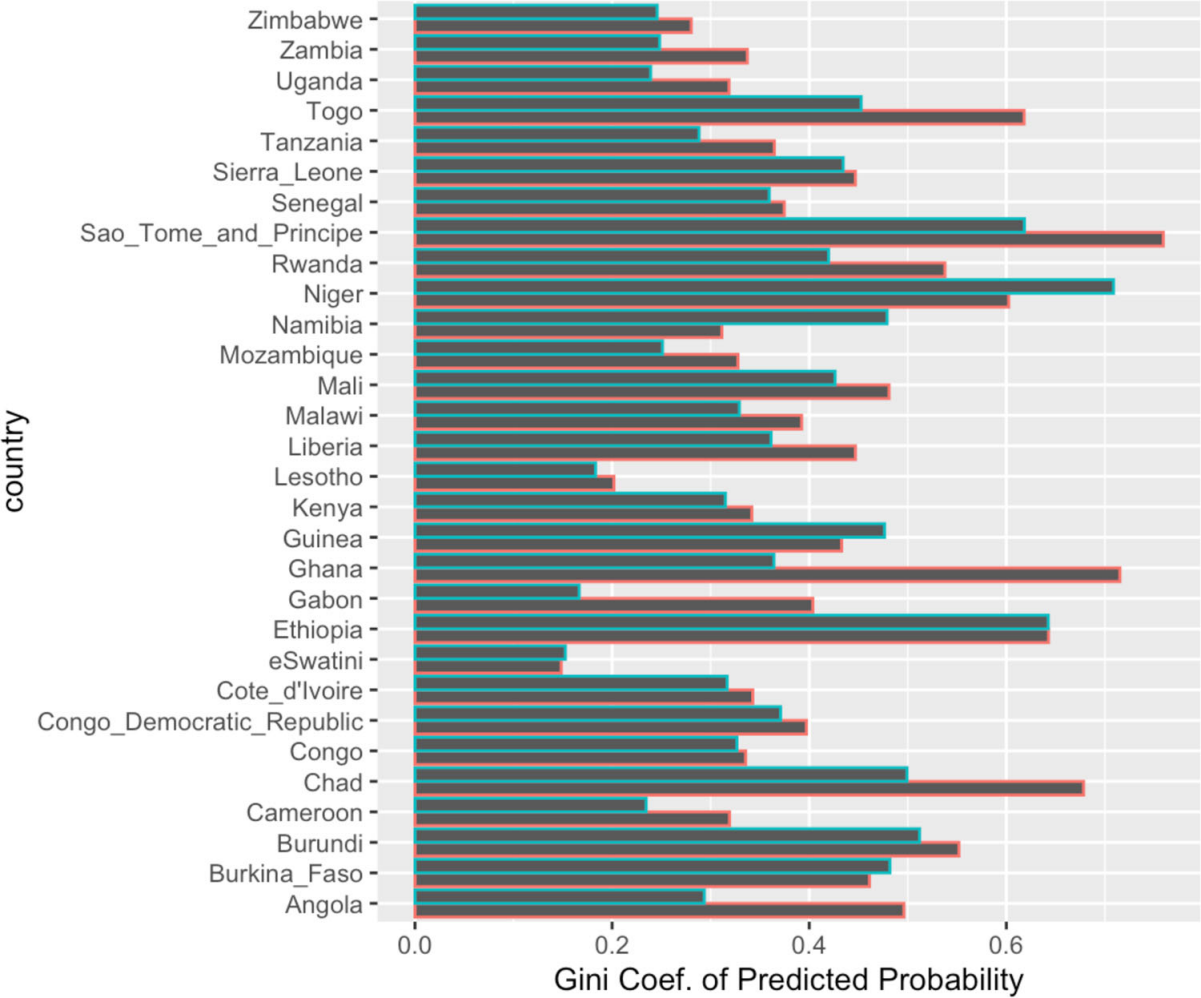
Concentration of HIV predicted risk (Gini coefficient). Gini coefficient of the predicted probability per country in Sub-Saharan Africa. Blue boxes are for males, red boxes are for females. Sample sizes for cases and controls for each Gini coefficient calculation are in Table S2.

The use of the top 10 variables improved prediction of HIV status relative to use of prevalence rates for risk prediction across every country in the study (Fig. 4A, B). For some countries (e.g. eSwatini) the predicted probability of HIV risk was fairly evenly distributed across the samples (Gini=0.15) (Fig. 5). On the other hand, the Gini coefficient was upwards of 0.5 in several countries (including Ethiopia, Niger, and females in Rwanda) (Fig. 5). Sample sizes of HIV+ and non-positive controls are in Supplementary Data 7.

## Discussion

Here, we use a data-driven approach for discovery of candidate behavioral, social, and economic risk factors for HIV at scale, and in doing so, present extensive correlates of HIV infection in Sub-Saharan Africa that span economic, biological, and environmental domains. Finally, we apply this approach for HIV risk prediction across the subcontinent, and show that risk assessment of HIV status can be improved using this non-invasive epidemiologic approach.

Several identified risk factors deserve further discussion because of their strong association with HIV status, their pervasive presence across the study countries, and their relatively large explanatory power. Being the head of the household stands out for identifying relatively large risk of HIV among females in the majority of our study countries. This is an intuitive correlate of risk, and possibly identifies women whose husbands had HIV and passed away, but its relative ubiquity has not been previously recognized. Complements of this risk factor – women living in households with a male head and who have been in a single marriage – are at lower risk of having HIV. Variables that characterize marital status commonly are also closely associated with HIV status. While our study is limited in its mechanistic insights into these associations, we note the possibility of multiple pathways, including behavioral pathways, that deserve further study given the strength of the associations.

Another important identified variable group relates to schooling and educational attainment among both males and females. Not attending school was a consistent correlate of increased risk among females, while completion of primary school was a protective correlate among men. Because school and educational attainment indicators are often closely correlated with one another, additional schooling correlates would be identified if our heuristic (for example, the one hot-encoded complement of attending school among females) did not eliminate them. Finding educational correlates that are in line with causal effect estimates among our top variables supports the importance of schooling in reducing HIV risk^21^.

Several less intuitive variables were also commonly associated with HIV. Non-ownership of livestock is a consistent positive correlate of HIV, among both males and females. We believe that this may be more closely associated with residence in urban environments (non-ownership of agricultural land also carries elevated risk of HIV among both males and females, in fewer surveys). Notably, we do not observe urban residence among the top candidate risk factors for HIV, suggesting livestock ownership carries additional (indirect) linkages to HIV risk.

Our adjusted analyses shed further light on confounding in our identified variables. We observe smaller non-zero association sizes (with small standard errors) among our top variables in the analyses adjusted for age, residence, and wealth. This suggests that our findings are likely at least partly confounded. That said, even very broad measures such as age, residence, and wealth fail to sever the association of nearly all of our identified variables. In the large majority of cases, the associations remain strong even in adjusted analyses. Confounding is important when considering causal relationships, as modifying a confounded variable is less likely to affect the outcome of interest. For the other goals of our analysis – identification of potential under-recognized risk factors, HIV status prediction, and targeting of public health services, among others – confounded but tightly associated relationships remain informative. Finally, we note that our adjusting variables – especially residence and wealth – are themselves complex constructs that may not be causal in a simple way. Identifying the components of these constructs – for example, livestock ownership as a component of wealth – may provide useful insights.

A notable advantage of our approach is its systematic assessment of the associations of all the variables assessed in DHS, creating for the first time a database of robust (e.g., reproducible association sizes across multiple waves) across all DHS-surveyed sub-Saharan Africa countries. In comparison to “candidate” association studies that examine a few or a handful of associations at a time, that a systematic approach may lead to more consistent positive, as well as negative, identification of important correlates. This highlights the importance of examining plausible pathways linking the identified variables to HIV: the consistency of the variables we identify lends them statistical strength, and that strength further benefits from real-world context.

A systematic approach also provides a database to contextualize associations. Male circumcision, originally identified in observational studies, is associated with a meta-analytic relative risk of 0.52 (an absolute value RR of roughly 2)^7^. This association size would rank in the top 80th percentile of pan-sub-Saharan Africa associations available in this report. In other words, ~1257 variables would meet this threshold among all variables assessed in men and a fraction of those that are robust across the continent (Fig. 1B). Further, we emphasize that the DHS aims to collect a representative sample of populations surveyed and we anticipate little sample selection bias among the potential database of correlates and HIV+.

Another strength of this large multi-country person-level analysis is the demonstration of HIV risk distribution in different sample populations. For example, we can measure the concentration of risk in different sample populations. We show large variation in HIV risk distribution between countries: HIV risk is highly concentrated among a small portion of the sample population in some countries, while risk is more distributed in the sample population in other countries. If all risk is concentrated in a small portion of the population, then identifying at-risk groups may be more feasible from a public health perspective than if risk is more evenly distributed across a large portion of the population. We also note that, in most countries, HIV risk is more concentrated in females than in males, and that HIV risk concentration is very high (upwards of 60%) in a handful of countries (Chad, Ghana, Togo, and Ethiopia).

There are limitations to our data-driven study. First, the associations that we identify emerge may be confounded. Second, we applied a stringent heuristic (Bonferroni pvalue and pseudo-R^2^ thresholds) for identification of associations from a massive database. Therefore, some associations may be “false negative” and fail to be discussed. We have provided all associations for readers to examine in broader context in the Supplementary Data 1–6. Third, our correlations are cross-sectional and we cannot rule out potential reverse-causal relationships. Fourth, aside from the HIV test, many variables are self-reported and may have differential measurement error rates. As we reported^11^ random error or non-differential bias will lead to reduction of association sizes toward the null.

In this extensive analysis of risk factors for HIV positivity, we are able to systematically characterize HIV risk factors, including identification of under-appreciated risk factors with meaningful association sizes, as well the extent to which risk factors are shared across countries and over time. This approach can be used to better characterize HIV risk using observational data, to identify hypotheses for HIV interventions, as well as serve as a platform for developing tools for identification risk of important non-HIV outcomes.

## Supplementary information

Description of Additional Supplementary Files

Supplementary Data 1

Supplementary Data 2

Supplementary Data 3

Supplementary Data 4

Supplementary Data 5

Supplementary Data 6

Supplementary Data 7

Reporting Summary

Supplementary Information

## Data Availability

The DHS datasets were downloaded as zipped Stata files and are available for download at https://dhsprogram.com. Up to six files were obtained per country-survey if they were available: household member recode (PR), individual recode (IR), men’s recode (MR), household recode (HR), HIV test results recode (AR), and geographic data (GE). All summary data and code files reported here (including supplementary data) can also be found on Zenodo (10.5281/zenodo.6819777)^20^. All survey summary statistics can be found on Zenodo, called “univariateResults_combined.zip” (10.5281/zenodo.6819777). The source data to recreate Figures and Tables can be found here: https://github.com/chiragjp/dhs_hiv_meta/blob/master/meta_data/meta_filtered_data.Rdata. See Code Availability for creation of this file.

## Code availability

The codebase for data preparation is available on Zenodo (10.5281/zenodo.6819777)^20^, in files called dhs-processing-master.zip, hiv-aws-master.zip. The source code for meta-analysis across surveys are available here: https://github.com/chiragjp/dhs_hiv_meta and have been mirrored in Zenodo (10.5281/zenodo.6819777: dhs_hiv_meta-master.zip)^20^. To recreate Figures and Tables, scripts in the *dhs_hiv_meta* repository are executed on the survey summary statistics, “univariateResults_combined.zip”. See the code in the *dhs_hiv_meta* repository located here to start: https://github.com/chiragjp/dhs_hiv_meta/blob/master/meta_pipeline_combined.R. A website of the findings is here: https://www.chiragjpgroup.org/dhs_hiv_meta/.

## References

[1] 90-90-90: treatment for all. http://www.unaids.org/en/resources/909090. (2022)

[2] UNAIDS data 2019. https://www.unaids.org/en/resources/documents/2019/2019-UNAIDS-data (2019)

[3] Serwadda D (1992). HIV risk factors in three geographic strata of rural Rakai District, Uganda. Aids.

[4] Simbayi (2005). Risk factors for HIV-AIDS among youth in Cape Town, South Africa. Aids Behav.

[5] Bulstra CA (2020). Mapping and characterising areas with high levels of HIV transmission in sub-Saharan Africa: A geospatial analysis of national survey data. PLOS Med..

[6] Gersovitz M (2005). The HIV epidemic in four African countries seen through the Demographic and Health Surveys. J. Afr. Econ..

[7] Weiss HA, Quigley MA, Hayes RJ (2000). Male circumcision and risk of HIV infection in sub-Saharan Africa: a systematic review and meta-analysis. Aids.

[8] Ioannidis JP (2005). Why most published research findings are false. PLoS Med..

[9] Ioannidis JP, Tarone R, McLaughlin JK (2011). The false-positive to false-negative ratio in epidemiologic studies. Epidemiology.

[10] Patel CJ, Ioannidis JP (2014). Studying the elusive environment in large scale. JAMA.

[11] Patel CJ, Bhattacharya J, Ioannidis JPA, Bendavid E (2018). Systematic Identification of Social, Behavioral, Environmental, and Economic Correlates of HIV Infection in Zambia in 2013–2014: An X-Wide Association Study. Aids.

[12] Staveteig, S. E., Bradley, S. E. K., Nybro, E. & Wang, S. *Demographic Patterns of HIV Testing Uptake in Sub-Saharan Africa. DHS Comparative Reports No. 30. ICF International*. (2013).

[13] Sorokin, A. & Forsyth, D. Utility data annotation with Amazon Mechanical Turk. in *2008 IEEE Computer Society Conference on Computer Vision and Pattern Recognition Workshops* 1–8 10.1109/CVPRW.2008.4562953 (2008).

[14] Borenstein, M., Hedges, L. V., Higgins, J. P. T. & Rothstein, H. R. *Introduction to Meta-Analysis*. (Wiley, 2011).

[15] Viechtbauer W (2010). Conducting Meta-Analyses in R with the metafor Package. J. Stat. Software.

[16] Hosmer, D., Lemeshow, S. & Sturdivant, R. X. *Applied Logistic Regression*. (John Wiley & Sons, 2013).

[17] Lumley T (2004). Analysis of complex survey samples. J. Stat. Software.

[18] ineq: Measuring Inequality, Concentration, and Poverty version 0.2-13 from CRAN. https://rdrr.io/cran/ineq/. (2022)

[19] Milanovic B (1997). A simple way to calculate the Gini coefficient, and some implications. Econ. Lett..

[20] Patel C. J. et al. Data and software for Sub-Saharan HIV+ x-wide associations. *Zenodo*10.5281/zenodo.6819777 (2022).

[21] Duflo E, Dupas P, Kremer M (2015). Education, HIV, and Early Fertility: Experimental Evidence from Kenya. Am. Econ. Rev..

