# Supplementary material for: The demographic and socioeconomic correlates of behavior and HIV infection status across sub-Saharan Africa
Source: Commun Med (Lond). 2022 Aug 18;2:104. doi: 10.1038/s43856-022-00170-z (PMC9388647; doi:10.1038/s43856-022-00170-z)
Supplement: Supplementary file 1 — Description of Additional Supplementary Files [file 43856_2022_170_MOESM1_ESM.pdf]

Description of Additional Supplementary Files

File Name: Supplementary Data 1
Description: Summary statistics (meta-analytic OR, Nagelkerke R2, and I2) for females across all surveys

File Name: Supplementary Data 2

Description: Summary statistics (meta-analytic OR, Nagelkerke R2, and I2) for males across all surveys

File Name: Supplementary Data 3
Description: Country-specific summary statistics for males.

File Name: Supplementary Data 4
Description: Country-specific summary statistics for females

File Name: Supplementary Data 5
Description: Multivariate model results for all variables appearing in greater than 10 countries for females

File Name: Supplementary Data 6

Description: Multivariate model results for all variables appearing in greater than 10 countries for females

File Name: Supplementary Data 7

Description: Sample Sizes for prediction of HIV+ per country
