# Supplementary material for: The demographic and socioeconomic correlates of behavior and HIV infection status across sub-Saharan Africa
Source: Commun Med (Lond). 2022 Aug 18;2:104. doi: 10.1038/s43856-022-00170-z (PMC9388647; doi:10.1038/s43856-022-00170-z)
Supplement: Supplementary file 10 — Supplementary Information [file 43856_2022_170_MOESM10_ESM.pdf]

The Demographic, Social, and Economic Correlates of HIV Infection Status in Sub-Saharan Africa

Supplementary Information

Chirag J. Patel, Kajal Claypool, Eric Chow, Jake Chung, Don Mai, Jessie Chen, Eran Bendavid

Supplementary Figures:

Figure S1. Number of Variables assessed as a function of number of surveys and countries.

Figure S2. Empirical CDF of (A) Nagelkerke R2, (B) exp(absolute value(beta)) or OR, and (C) Heterogeneity (I2). Red line depicts CDF for those not identified, blue line identified (e.g., pvalue < 1e-6 and R2 > 0.001).

Figure S3. Comparison of effect size between univariate and multivariate models

Supplementary Table:

Table S1. Distributions of odds ratios, Nagelkerke R^2, and I^2 (heterogeneity) estimates for variables appearing in 1 country and at least 2 countries.

Supplementary Figures

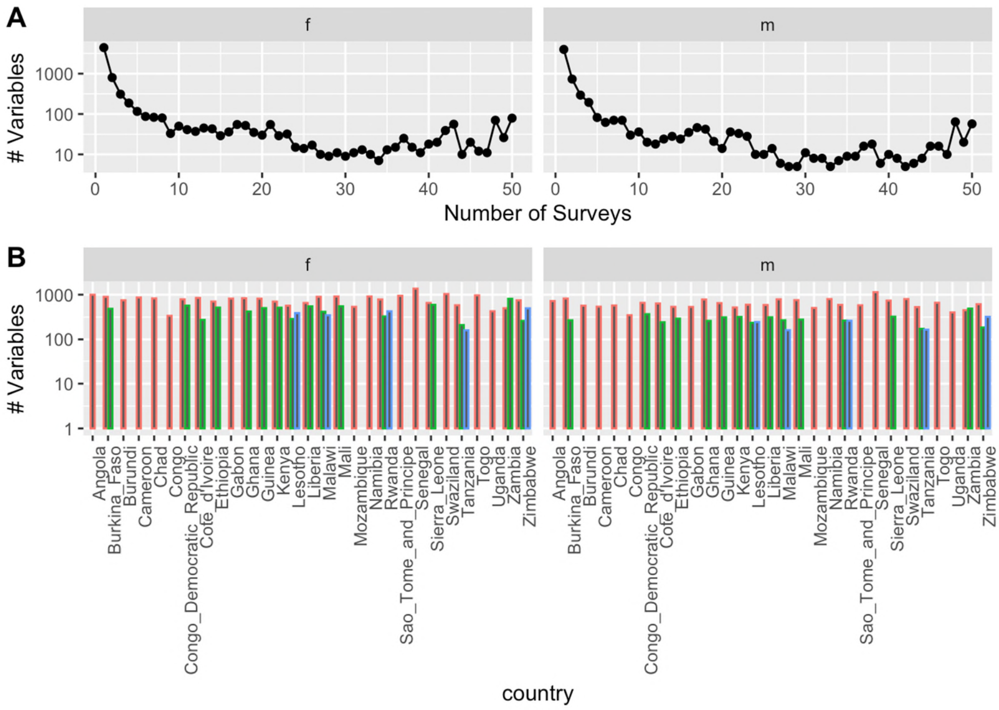

Figure S1. Number of Variables assessed as a function of number of surveys and countries.

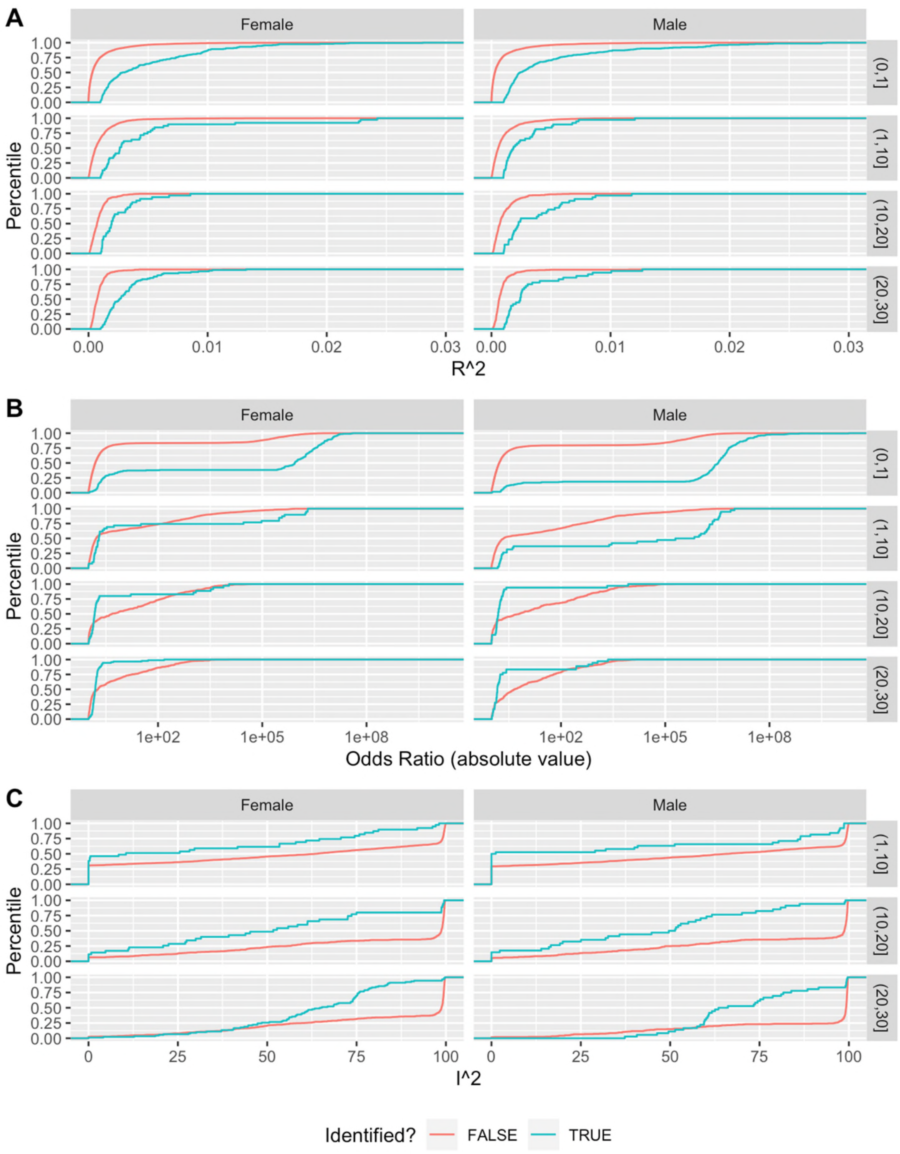

Figure S2. Empirical CDF of (A) Nagelkerke R2, (B) exp(absolute value(beta)) or OR, and (C) Heterogeneity (I2). Red line depicts CDF for those not identified, blue line identified (e.g., pvalue < 1e-6 and R2 > 0.001).

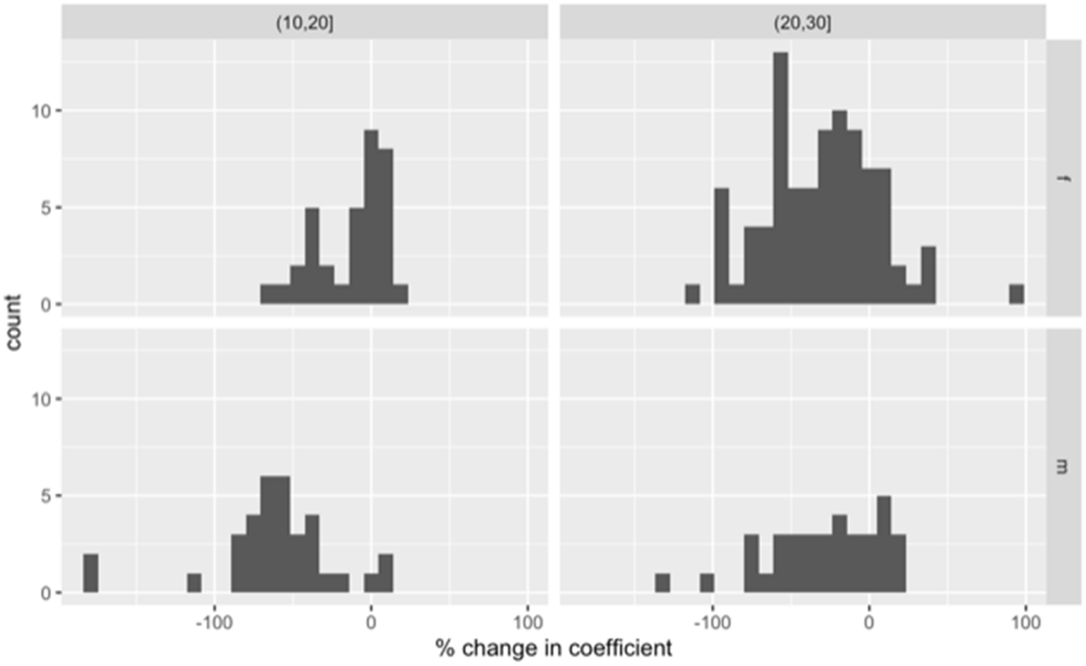

Figure S3. Comparison of association size between univariate and multivariate models as percent difference of the multivariate adjusted associate versus the univariate association. (beta(adjusted) – beta(univariate) / beta(univariate) * 100). Associations that were significant in the univariate analysis are shown. m denotes males; f denotes females. (10,20] and (20,30] denotes the number of countries a variable appeared in.

gender/number	Num Associations	25th OR	Median OR	75th OR
**1 Survey**				
f	4543	1.2	1.62	3.69
m	4089	1.24	1.78	12.67
**>1 Surveys**				
f	2708	1.18	1.73	73.83
m	2199	1.24	2.6	263.09

Table S1. Distributions of odds ratios, and I^2 (heterogeneity) estimates for variables appearing in 1 country and at least 2 countries.

Table S2. Sample Sizes for used in prediction and Gini index calculation.

Country	Sex	N Cases	N Controls
Angola	f	25	1336
Angola	m	38	1632
Burkina_Faso	f	75	5418
Burkina_Faso	m	36	2170
Burundi	f	30	922
Burundi	m	47	2284
Cameroon	f	106	1706
Cameroon	m	147	2651
Chad	f	37	2318
Chad	m	24	880
Congo	f	44	1217
Congo	m	85	2675
Congo_Democratic_Republic	f	71	4386
Congo_Democratic_Republic	m	65	6477
Cote_d'Ivoire	f	173	2816
Cote_d'Ivoire	m	146	3149
eSwatini	f	290	604
eSwatini	m	369	622
Ethiopia	f	68	4752
Ethiopia	m	115	4325
Gabon	f	78	1500
Gabon	m	145	3063
Ghana	f	26	1859
Ghana	m	70	3289
Guinea	f	82	3706
Guinea	m	51	2419
Kenya	f	100	1232
Kenya	m	208	2609
Lesotho	f	548	1558
Lesotho	m	677	1508
Liberia	f	56	3060
Liberia	m	40	2070
Malawi	f	395	3372
Malawi	m	1008	7507
Mali	f	50	4001
Mali	m	43	3682

Mozambique	f	175	1090
Mozambique	m	293	1786
Namibia	f	124	625
Namibia	m	80	496
Niger	f	14	2113
Niger	m	11	426
Rwanda	f	111	2597
Rwanda	m	346	8758
Sao_Tome_and_Principe	f	3	423
Sao_Tome_and_Principe	m	12	391
Senegal	f	28	3437
Senegal	m	24	1908
Sierra_Leone	f	62	3997
Sierra_Leone	m	63	4259
Tanzania	f	385	7370
Tanzania	m	529	8364
Togo	f	16	1456
Togo	m	33	993
Uganda	f	207	3088
Uganda	m	369	4311
Zambia	f	814	4975
Zambia	m	1528	7630
Zimbabwe	f	879	3710
Zimbabwe	m	1519	5903
